# Identification of Off-Patent Compounds That Present Antifungal Activity Against the Emerging Fungal Pathogen *Candida auris*

**DOI:** 10.3389/fcimb.2019.00083

**Published:** 2019-04-02

**Authors:** Haroldo Cesar de Oliveira, Maria Candida Monteiro, Suélen Andreia Rossi, Javier Pemán, Alba Ruiz-Gaitán, Maria José Soares Mendes-Giannini, Emilia Mellado, Oscar Zaragoza

**Affiliations:** ^1^Mycology Reference Laboratory, National Centre for Microbiology, Instituto de Salud Carlos III, Madrid, Spain; ^2^Laboratório de Micologia Clínica, Departamento de Análises Clínicas, Faculdade de Ciências Farmacêuticas, Universidade Estadual Paulista (UNESP), Araraquara, Brazil; ^3^Hospital Universitari i Politécnic La Fe, Valencia, Spain; ^4^Instituto de Investigación Sanitaria La Fe, Valencia, Spain

**Keywords:** *Candida auris*, antifungals, drug repurposing, synergism, multiresistance

## Abstract

*Candida auris* is an emerging fungal pathogen of great concern among the scientific community because it is causing an increasing number of hospital outbreaks of difficult management worldwide. In addition, isolates from this species frequently present reduced susceptibility to azole and echinocandin drugs. For this reason, it is necessary to develop new antifungal strategies to better control the disease caused by this yeast. In this work, we screened drugs from the Prestwick chemical library, which contains 1,280 off-patent compounds that are already approved by the Food and Drug Administration, with the aim of identifying molecules with antifungal activity against *C. auris*. In an initial screening, we looked for drugs that inhibited the growth of three different *C. auris* strains and found 27 of them which it did so. Ten active compounds were selected to test the susceptibility profile by using the EUCAST protocol. Antifungal activity was confirmed for seven drugs with MICs ranging from 0.5 to 64 mg/L. Some of these drugs were also tested in combination with voriconazole and anidulafungin at sub-inhibitory concentrations. Our results suggest synergistic interactions between suloctidil and voriconazole with fractional inhibitory concentration index (FICI) values of 0.11 to 0.5 and between ebselen and anidulafungin (FICI, 0.12 to 0.44). Our findings indicate that drug repurposing could be a viable alternative to managing infections by *C. auris*.

## Introduction

Infections caused by fungi are an increasing threat for immunosuppressed patient, and their incidence has risen in the last few decades (Low and Rotstein, 2011; Brown et al., 2012; Pilmis et al., 2016). Invasive fungal diseases (IFDs) have a high associated mortality rate and economic cost (Dignani, 2014; Drgona et al., 2014). The fungal pathogens with a higher prevalence in clinical settings belong to *Candida* and *Aspergillus* genera (Sanglard, 2016). However, the changing epidemiology of the fungal pathogenic species is one of the main challenges in clinical mycology. This variation is mainly caused by the massive use of antifungals, especially azoles and echinocandins, which have caused the emergence of species with reduced susceptibility to these antifungals (such as *Candida krusei, C. glabrata, C. parapsilosis, A. terreus, Fusarium, Scedosporium*, and *Lomentospora*) (Arendrup and Perlin, 2014; Berkow and Lockhart, 2017).

Another case that illustrates the epidemiological challenge of IFDs is the recent emergence of *Candida auris* as pathogen. This species is an ascomycete, closely related to *C. haemulonii* and *C. lusitaniae* (Berkow and Lockhart, 2017; Lockhart et al., 2017a), and has been rarely reported as a causative agent of IFDs. But in the last 5 years, this fungus has caused multiple hospital outbreaks worldwide (Calvo et al., 2016; Schelenz et al., 2016; Sharma et al., 2016; Chow et al., 2018; de Cassia Orlandi Sardi et al., 2018; Kohlenberg et al., 2018; Ruiz-Gaitan et al., 2018). The control of these outbreaks has been difficult for several reasons. First, this yeast can easily be misidentified as *C. haemulonii* or *C. parapsilosis* in laboratories that do not perform identification through molecular biology or MALDITOF techniques (Chatterjee et al., 2015; Chowdhary et al., 2017). Another concern is the difficulty to control and eradicate the outbreaks from the affected areas. Even rapid patient to patient transmission has been reported (Schwartz and Hammond, 2017). For these reasons, some of these outbreaks have lasted several months or even years until they have been eliminated from the hospitals (Eyre et al., 2018; Ruiz-Gaitan et al., 2018). Concerning *C. auris* antifungal susceptibility profile, most isolates are intrinsically resistant to fluconazole, and there is also a high resistance rate reported to echinocandins and amphotericin, limiting the treatment options that can be administered to patients (Lockhart et al., 2017b; Sears and Schwartz, 2017; Kordalewska et al., 2018).

The treatment of fungal infections is frequently limited by the appearance of intrinsic and secondary resistance and to the low number of antifungal families available. This is more relevant to *C. auris* infection treatment, requiring the design of new therapeutic strategies. In the last few years, drug repurposing of “off-patent” drugs has become a feasible alternative to developing new treatments for invasive fungal diseases. This strategy offers important advantages compared to the development of new drugs, such as lower costs and shorter time required to implement use. In the case of fungal pathogens, drug repurposing has been successfully used to identify off-patent compounds against *C. albicans* and *Cryptococcus neoformans* (Butts et al., 2013; Siles et al., 2013; Kim et al., 2015; Wiederhold et al., 2017; Nixon et al., 2018). In this work, we have attempted to identify new drugs that present antifungal activity against *C. auris*. We have screened the Prestwick Chemical Library, which contains 1,280 off-patent compounds approved by the FDA for the treatment of many different diseases. We found several compounds that could be considered as alternative options to inhibit the growth of *C. auris* alone or in combination with antifungals, confirming that the repurposing of off-patent drugs is a promising approach to finding antimicrobial molecules.

## Materials and Methods

### Isolates and Culture Conditions

In this work we used five *C. auris* strains isolated in Spain and kept at Mycology Reference Laboratory: CL-10093, CL-9998, CL-10021, CL-10013, and CL-10030; one *C. auris* from Japan: JCM 15448 (Satoh et al., 2009); and another strain isolated from Korea: KCTC 17810 (Kim et al., 2009). All the isolates were propagated in agar Saboraud plates incubated at 30°C.

### Antifungal Susceptibility Testing

Antifungal susceptibility testing was performed using the broth microdilution method described by the European Committee on Antimicrobial Susceptibility Testing, EUCAST (E.Def 7.3.1 reference method) (Arendrup et al., 2016). The antifungal agents tested and the range of concentrations used were: amphotericin B (AmB) (0.03–16 mg/L) (Sigma Aldrich Quimica, Madrid, Spain), flucytosine (5FC) (0.12–64 mg/L) (ICN Pharmaceuticals, Orsay, France), fluconazole (FCZ) (0.12–64 mg/L) (Pfizer SA, Madrid, Spain), itraconazole (ITZ) (0.015–8 mg/L) (Janssen Pharmaceutical S.A., Madrid, Spain), voriconazole (VCZ) (0.015–8 mg/L) (Pfizer, S.A., Madrid, Spain), posaconazole (PSZ) (0.015–8 mg/L) (Schering-plow, Madrid, Spain), isavuconazole (IVZ) (0.015–8 mg/L) (Astellas Pharma Inc., Tokyo, Japan), caspofungin (CAS) (0.03–16 mg/L) (Merck Research Laboratories, Rahway, N.J), micafungin (MCF) (0.004–2 mg/L) (Astellas Pharma Inc., Tokyo, Japan), and anidulafungin (ANID) (0.007–4 mg/L) (Merck & Com, Inc, NJ, USA). *Candida auris* isolates were cultivated in agar Sabouraud plates at 35°C for 24 h and an inoculum at 1–5 × 10^5^ cells/mL was prepared in distillated water. Then, 100 μL of this inoculum were added to the library plates (final inoculum = 0.5–2.5 × 10^5^ cells/mL). The plates were incubated at 35°C and growth inhibition was evaluated after 24 h of incubation by measuring the optical density at 530 nm in a spectrophotometer. The minimal inhibitory concentration (MIC) was defined as the concentration that inhibited at least 50% of *C. auris* growth, except for AmB, for which MIC evaluated 90% of growth inhibition.

### Screening for Active Drugs Against *C. auris*

The Prestwick Library was purchased from Prestwick Chemical. The library is composed of 1,280 off-patent drugs in 96-well plates approved by the Food and Drug Administration (FDA) and European Medicines Agency (EMA). Each compound was prepared at 10 mM in dimethyl sulfoxide (DMSO). For the screening, each drug from the library was diluted to a final concentration of 50 μM in RPMI medium containing 2% glucose buffered at pH 7.0 with 165 mM MOPS. In each plate, growth and sterility controls were added. *Candida auris* inocula were suspended in distilled water as described above. The plates were incubated for 24 h at 35°C without shaking and the growth was evaluated by spectrophotometric readings at 530 nm. Active compounds against *C. auris* were determined as the ones that inhibited at least 50% of growth. This primary screening was performed using three *C. auris* strains with different geographical origin: CL-10093, JCM 15448, and KCTC 17810.

### Minimal Inhibitory Concentration of Active Drugs Against *C. auris*

The MIC of active compounds against *C. auris* was determined following EUCAST methodology (Arendrup et al., 2016). Antifungal susceptibility plates were prepared with a range of concentrations from 64 to 0.12 mg/L of each compound in RPMI. *C. auris* inocula were prepared as described using the same concentration to inoculate the plates. The plates were incubated at 35°C and growth inhibition was evaluated after 24 h of incubation by spectrophotometrical readings at 530 nm. The MICs were defined as the concentrations that inhibit at least 50% (MIC_50_) or 90% (MIC_90_) of fungal growth.

### Checkerboard Assay

The synergism of three active compounds (suloctidil, ciclopirox ethanolamine, and ebselen) with VCZ and ANID was also tested using the checkerboard assay. For this purpose, we used the following drug concentration ranges: the identified compounds ranged from 64 to 0.12 mg/L; VCZ ranged from 8 to 0.12 mg/L; and ANID ranged from 0.5 to 0.007 mg/L. To prepare the plate with the different drug combinations, 50 μL from each compound concentration were mixed with 50 μL of each antifungal concentration. Because the drugs and antifungal stocks were dissolved in DMSO, we ensured that, the DMSO concentration did not exceed 1% in the assay plate. Checkerboard plates were inoculated with 100 μL *C. auris* inoculum prepared as described above. Plates were incubated for 24 h at 35°C without shaking. Yeast growth was measured by spectrophotometric reading at 530 nm. The Fractional Inhibitory Concentration index (FICI) was calculated according to the equation: ΣFIC = FIC (Compounds) + FIC (VCZ or ANID). The FIC index represents the sum of the FICs of each drug tested, where the FIC is determined for each drug by dividing the MIC of each drug when used in combination by the MIC of each drug when used alone (Meletiadis et al., 2010). We considered a synergistic effect when ΣFIC was below 0.5 (White et al., 1996).

## Results

### Antifungal Susceptibility Profile of *C. auris* Isolates Against Antifungal Drugs

We first characterized the *in vitro* antifungal susceptibility of three *C. auris* strains from different geographical origins. As shown in Table 1, all the strains had reduced susceptibility to all azoles tested, which was more prominent for FCZ and VCZ. In the case of FCZ, all the isolates were fully resistant to this antifungal (≥64 mg/L). These isolates were susceptible to echinocandins, although these antifungals did not fully inhibit the growth of the yeasts. Echinocandins presented a significant trailing growth, which was more prominent after 48 h of incubation (data not shown). All the strains had low MIC values to 5FC (Table 1) and AmB.

**Table 1:** Antifungal susceptibility test to three *C. auris* strains.

**Antifungal drugs**	**MIC values (mg/L) for the** ***C. auris*** **strains**		
	**CL-10093**	**JCM 15448**	**KCTC 17810**
Amphotericin B (AmB)	0.12	0.12	0.03
Flucytosine (5FC)	0.12	0.12	0.25
Fluconazole (FCZ)	>64	64	>64
Itraconazole (ITZ)	0.12	0.12	0.5
Voriconazole (VCZ)	4	4	2
Posaconazole (PSZ)	0.12	0.06	0.5
Isavuconazole (IVZ)	0.06	0.03	2
Caspofungin (CAS)	0.5	0.5	0.5
Micafungin (MCF)	0.125	0.125	0.125
Anidulafungin (AND)	0.03	0.03	0.06

### Identification of Active Compounds Against *C. auris*

Three clinical *C. auris* strains (CL-10093, KCTC 17810, and JCM 15448), were used to identify active compounds among the 1,280 compounds from the Prestwick Chemical Library. In this initial screening, we identified 27 active compounds belonging to different classes. Twelve of them inhibited growth ≥90% of the three *C. auris* strains and 15 inhibited growth more than 50% (Table 2). Among them, we found compounds with different therapeutic effects such as antibacterial, antineoplastic, antihypertensive, antidepressant, anticonvulsant, antiplatelet, anti-inflammatory, antipsychotic, and antipruritic. We also identified 13 antifungal drugs belonging to distinct antifungal classes that were active against the three *C. auris* strains (Table 3).

**Table 2:** Active compounds at 0.05 mM against *C. auris* strains CL-10093, JCM 15448, and KCTC 17810.

**Name**	**mg/L**	**Therapeutic effect**	Target/Action mechanism^*^	**Growth inhibition (%)**		
				**CL-10093**	**JCM 15448**	**KCTC 17810**
Chlorhexidine	25	Antibacterial	Reacts with the negatively charged microbial cell surface destroying the integrity of the cell membrane. Also penetrates into the cell and causes leakage of intracellular components leading to cell death.	98	99	98
Tamoxifen citrate	28	Antineoplastic	Act as an anti-estrogen in the mammary tissue, but as an estrogen in cholesterol metabolism, bone density, and cell proliferation in the endometrium.	98	98	90
Chloroxine	10	Antibacterial	Although the mechanism of action is not fully understood, topical administration diminishes mitotic activity in the epidermis, reducing excessive scaling associated with dandruff or seborrheic dermatitis of the scalp.	95	97	98
Ciclopirox ethanolamine	13	Antibacterial/Antifungal	Exerts its action by binding to and chelating trivalent cations inhibiting the availability of essential co-factors for enzymes. This may lead to a loss of activity of enzymes that are essential for cellular metabolism, organization of cell wall structure, and other crucial cell functions.	94	97	94
Methyl benzethonium chloride	23	Antibacterial	Adsorb onto the negatively charged cell wall of microorganisms, interrupting normal cell metabolism, leading to cell death, or growth inhibition.	98	97	100
Guanadrel sulfate	10	Antihypertensive	Postganglionic adrenergic blocking agent.	97	97	97
Alexidine dihydrochloride	29	Antibacterial	Potent and selective PTPMT1 (Protein Tyrosine Phosphatase Localized to the Mitochondrion 1) inhibitor.	97	97	97
Rolipram	13	Antidepressant	Phosphodiesterase 4 inhibitor with antidepressant properties.	98	97	91
Thonzonium bromide	29	Antiseptic	Monocationic surface-active agent with surfactant and detergent properties.	98	98	98
(-)-MK 801 hydrogen maleate	16	Anticonvulsant	N-methyl-D-aspartate receptor antagonist that acts at the NMDA receptor-operated ion channel as an open channel blocker, preventing Ca^2+^ flux.	98	98	97
Benzethonium chloride	22	Antibacterial	Adsorb onto the negatively charged cell wall of microorganisms, interrupting normal cell metabolism, leading to cell death or growth inhibition.	96	97	100
Suloctidil	16	Antiplatelet	It is not clear the mechanism of action of Suloctidil, but it might act either as inhibitor of thromboxane synthase or as a thromboxane receptor antagonist.	97	99	78
Ebselen	13	Anti-Inflammatory	Acts as a glutathione peroxidase mimetic and is thereby able to prevent cellular damage induced by reactive oxygen species (ROS).	87	91	92
Thiethylperazine dimalate	19	Antiemetic	Act as a dopamine antagonist.	68	90	86
Trifluoperazine dihydrochloride	24	Antiemetic	Act as a dopamine antagonist.	54	88	61
Pyrvinium pamoate	19	Anthelmintic	Interfere with glucose uptake by pinworms, is also thought to inhibit mitochondrial respiration complex 1 and suppress the unfolded protein response.	40	76	61
Clioquinol	15	Antiamebic/Antibacterial	Bacteriostatic compound, however, the precise mechanism of its action is unknown.	89	93	93
Hexachlorophene	20	Antiseptic	Inhibit the membrane-bound part of the electron transport chain, respiratory D-lactate dehydrogenase. It induces leakage, causes protoplast lysis, and inhibits respiration.	97	98	86
Dequalinium dichloride	26	Antibacterial	Disrupts bacteria cell permeability and also binds to the cytoplasmic membrane with subsequent formation of complexes and protein precipitation that lyses the membrane, perturbing osmotic exchange.	81	86	89
Methiothepin maleate	23	Antipsychotic	Act as a serotonin receptor antagonist.	74	72	64
Dyclonine hydrochloride	16	Local anesthetic	Binds to activated sodium channels on the neuronal membrane, decreasing the neuronal membrane's permeability to sodium ions, leading to an increased threshold for excitation.	63	63	54
Fipexide hydrochloride	21	Anti-fatigue	Interacts with the polar heads in the phospholipids membrane influencing in membrane function and fluidity.	68	63	54
Prochlorperazine dimaleate	30	Antiemetic/Antipsychotic	Act as D2 receptor antagonist.	71	66	72
Artemisinin	14	Antimalarial	Act by generating free radicals that in turn damage susceptible proteins, resulting in the death of the parasite	65	56	61
Dimethisoquin hydrochloride	15	Antipruritic	Inhibits nicotinic acetylcholine receptors with the maximum inhibition potency occurring for the α4β4 subtype.	59	65	51
Sertraline	15	Antidepressant	Inhibit the reuptake of serotonin at the presynaptic membrane.	59	88	56
Zotepine	16	Antipsychotic	Act as a dopamine antagonist that has a high affinity for D1- and D2-like receptors.	59	63	56

* *Information about the action mechanisms has been extracted from the information provided by Prestwick Chemical, and from DrugBank (https://www.drugbank.ca), and PubChem (https://pubchem.ncbi.nlm.nih.gov)*.

**Table 3:** Antifungal agents active against *C. auris* strains CL-10093, JCM 15448, and KCTC 17810.

**textbfName**	**mg/L**	Target/Action mechanism^*^	**Growth inhibition (%)**		
			**CL-10093**	**JCM 15448**	**KCTC 17810**
Amphotericin B	46	Binds to ergosterol, an essential component of the fungal cell membrane, causing depolarization of the membrane and altering cell membrane permeability.	97	97	95
Nystatine	46		97	98	97
Haloprogin	18	The mechanism of action is unknown, but it is thought to be via inhibition of oxygen uptake and disruption of yeast membrane structure and function.	96	96	83
Ketoconazole	26		88	92	97
Voriconazole	17		87	86	95
Clotrimazole	17		89	93	78
Tioconazole	19	Inhibit cytochrome P450 14-alpha-demethylase, which leads to a decrease in ergosterol concentration leading to disrupts in the structure and function of the fungal cell.	86	84	77
Terconazole	26		88	92	97
Econazole nitrate	19		72	82	50
Itraconazole	35		63	68	71
Sertaconazole nitrate	25		67	92	56
Hexetidine	16		98	98	53
Flucytosine	6	After penetration into the fungal cells, flucytosine is deaminated to its active metabolite 5-fluorouracil. 5-fluorouracil replaces uracil during fungal RNA synthesis, thereby inhibiting fungal protein synthesis.	98	95	94

* *Information about the action mechanisms has been extracted from the information provided by Prestwick Chemical, and from DrugBank (https://www.drugbank.ca), and PubChem (https://pubchem.ncbi.nlm.nih.gov)*.

### Antifungal Susceptibility Testing of the Selected Compounds

Ten active compounds were selected to analyze their activity against seven *C. auris* isolates using EUCAST protocol. We confirmed antifungal activity for 7 compounds: trifluoperazine dihydrochloride, suloctidil, ciclopirox ethanolamine, ebselen, tamoxifen citrate, thiethylperazine dimalate, and pyrvinium pamoate. For three compounds [(-)-MK 801 hydrogen maleate, rolipram, and guanadrel sulfate] antifungal activity was not reproduced (Table 4). We did not find noticeable differences in the susceptibility to the compounds when both MIC_50_ and MIC_90_ were calculated and compared.

**Table 4:** MIC_50_ and MIC_90_ for the active compounds against *C. auris* strains.

	**MIC values (mg/L)**													
	**CL-10093**		**KCTC 17810**		**JCM 15448**		**CL-10021**		**CL-9998**		**CL-10030**		**CL-10013**	
	**MIC_**50**_**	**MIC_**90**_**	**MIC_**50**_**	**MIC_**90**_**	**MIC_**50**_**	**MIC_**90**_**	**MIC_**50**_**	**MIC_**90**_**	**MIC_**50**_**	**MIC_**90**_**	**MIC_**50**_**	**MIC_**90**_**	**MIC_**50**_**	**MIC_**90**_**
(-)-MK 801 hydrogen maleate	>64	>64	>64	>64	>64	>64	>64	>64	>64	>64	>64	>64	>64	>64
Trifluoperazine dihydrochloride	32	64	32	64	16	32	32	64	32	64	32	64	64	64
Suloctidil	8	8	8	16	4	4	8	8	8	8	4	8	8	8
Ciclopirox ethanolamine	1	4	0.5	2	0.5	1	1	4	1	8	1	1	1	1
Ebselen	4	4	4	4	2	4	4	4	2	4	2	4	4	8
Tamoxifen citrate	16	32	32	64	16	16	32	32	16	32	16	32	16	32
Rolipram	>64	>64	>64	>64	>64	>64	>64	>64	>64	>64	>64	>64	>64	>64
Thiethylperazine dimalate	64	64	64	64	16	32	32	64	32	64	32	64	64	64
Guanadrel sulfate	>64	>64	>64	>64	>64	>64	>64	>64	>64	>64	>64	>64	>64	>64
Pyrvinium pamoate	4	4	2	4	1	2	2	4	2	4	2	4	2	4

### Synergism Effect of Suloctidil, Ebselen, and Ciclopirox Ethanolamine With Voriconazole and Anidulafungin

Three compounds—suloctidil, ebselen, and ciclopirox ethanolamine—were selected to test synergism with antifungal drugs of clinical use. We did not choose FCZ because the isolates were fully resistant to this drug. VCZ was chosen because, although having high MIC values against *C. auris*, it still had some *in vitro* activity. In parallel, synergism with ANID was also tested because echinocandins have become the first treatment option for invasive candidiasis. The three compounds (64–0.12 mg/L) were combined with VCZ (8–0.12 mg/L) and ANID (0.5–0.007 mg/L). The same seven isolates used in the MICs assays were tested in the checkerboard assay. Synergism was evaluated at 24 h and the FIC was calculated for 50 and 75% of growth inhibition.

**Figure 1:**
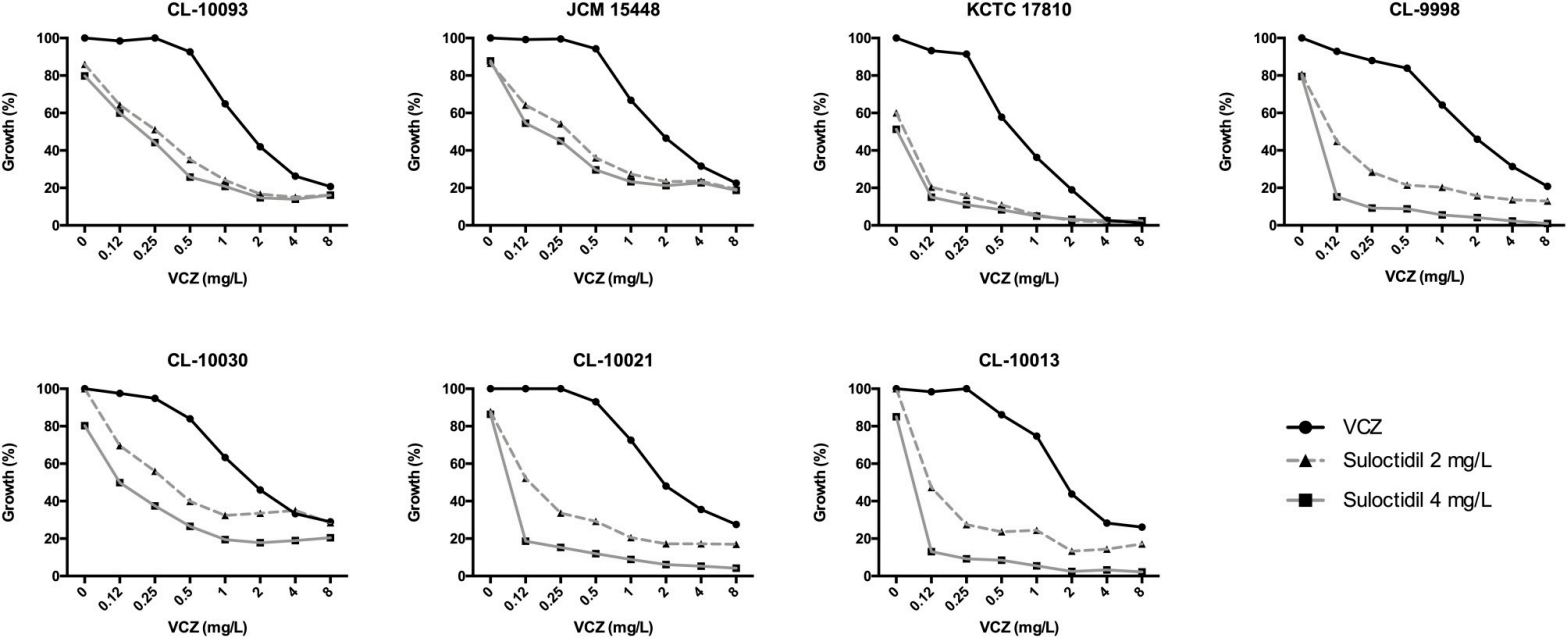
Representative graphs of the synergistic effect of VCZ (ranging concentration: 8–0.12 mg/L) in combination with 2 and 4 mg/L of suloctidil against *C. auris* strains by checkerboard assay.

We found that the combination of suloctidil and VCZ was synergistic against *C. auris* (FICI values < 0.5 for both 50% and 75% of growth inhibition, Table 5). The MICs for VCZ alone were around 2–4 mg/L, and in combination with different suloctidil concentrations the MICs values decreased to 0.12–1 mg/L (Figure 1).

**Table 5:** Fractional Inhibitory Concentration Index for the combination of voriconazole with suloctidil against *C. auris*.

***C. auris* strains**	**FICI**	
	**50%**	**75%**
CL-10093	0.37	0.40
JCM 15448	0.34	0.00
KCTC 17810	0.31	0.11
CL-9998	0.25	0.31
CL-10013	0.37	0.50
CL-10021	0.37	0.31
CL-10030	0.37	0.28

**Figure 2:**
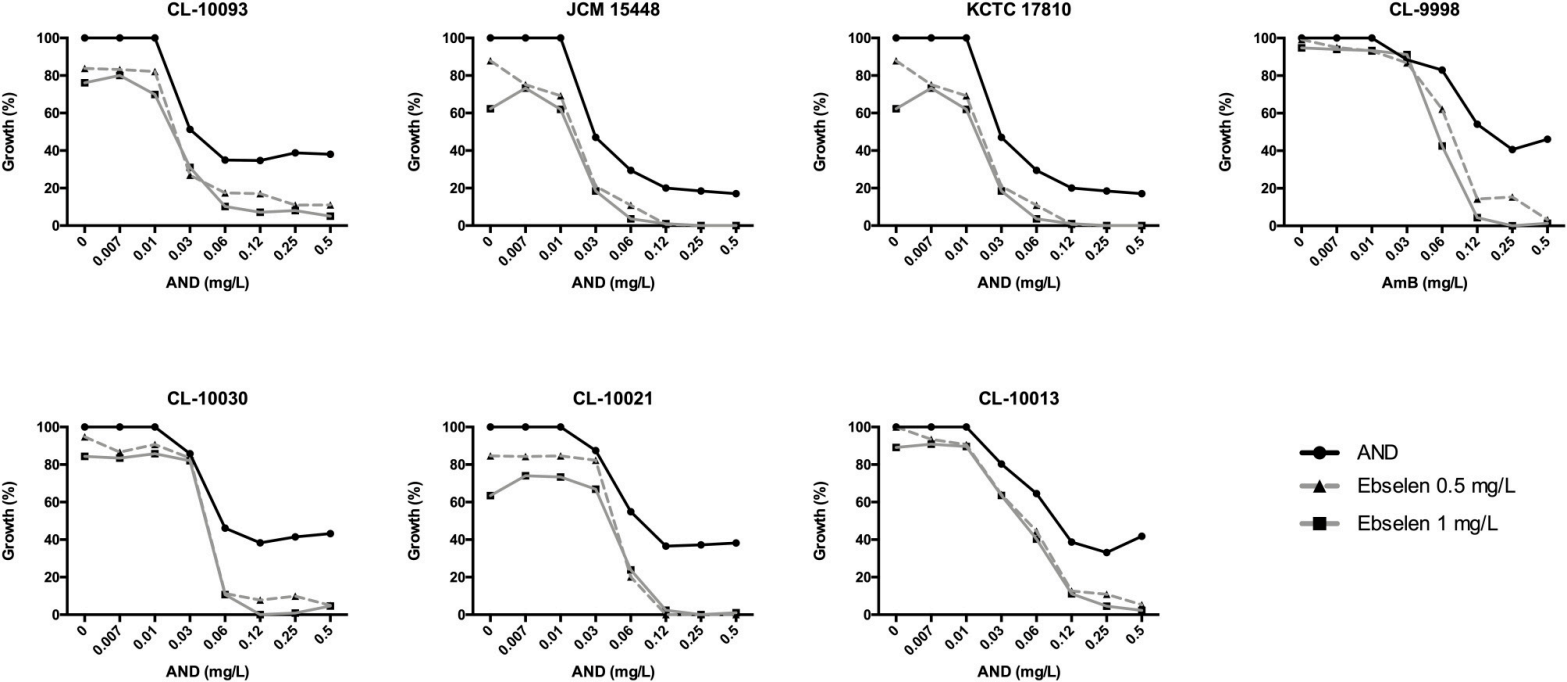
Representative graphs of the synergistic effect of AND (ranging concentration: 0.007–0.5 mg/L) in combination with 0.5 and 1 mg/L of ebselen against *C. auris* strains by checkerboard assay.

In addition, the combination of ebselen and ANID was also synergistic against this fungal pathogen (FICI values < 0.5 for 75% of growth inhibition) (Table 6). *C. auris* isolates have ANID MIC values of 0.12 mg/L. However, when combined with 0.5 or 1 mg/L of ebselen, we observed an increase in the susceptibility of all the *C. auris* isolates tested, reaching almost 90% of growth inhibition (Figure 2).

**Table 6:** Fractional Inhibitory Concentration Index for the combination of anidulafungin with ebselen against *C. auris*.

***C. auris* strains**	**FICI**	
	**50%**	**75%**
CL-10093	0.82	0.12
JCM 15448	1.13	0.28
KCTC 17810	1.64	0.44
CL-9998	0.50	0.25
CL-10013	0.56	0.25
CL-10021	1.03	0.25
CL-10030	1.03	0.12

No synergism was found when ciclopirox ethanolamine was combined with VCZ or AND.

## Discussion

The first isolation and characterization of *C. auris* as pathogen was in 2009 when it was isolated from an external ear canal drainage from a patient in Japan (Satoh et al., 2009). However, retrospective studies have demonstrated that this yeast has been the causative agent of diseases since 1996 (Lee et al., 2011). The frequency of detection of *C. auris* has increased in recent years, revealing a rapid and worldwide emergence of this pathogen (Satoh et al., 2009; Lee et al., 2011; Navalkele et al., 2017; Bidaud et al., 2018; Eyre et al., 2018; Kohlenberg et al., 2018; Ruiz-Gaitan et al., 2018) with a strong potential for nosocomial transmission added to a great capacity for developing multidrug antifungal resistance (Colombo et al., 2017).

The treatment of *C. auris* infections represents a great challenge due to its antifungal susceptibility profile. The majority of *C. auris* strains have high MIC values FCZ (>64 mg/L), suggesting intrinsic resistance to this drug (Chowdhary et al., 2017). A great number of isolates also exhibits elevated MICs to VCZ and a few isolates may also be considered resistant to echinocandins (Chowdhary et al., 2016, 2017; Colombo et al., 2017). The mechanisms of antifungal resistance in *C. auris* remain unknown, although increased tolerance to antifungal drugs may be partly explained because a significant portion of *C. auris* genome encodes transporters belonging to the ABC and major facilitator superfamilies (Chatterjee et al., 2015). Furthermore, whole genome sequencing of multiple isolates from different geographical regions have demonstrated that the majority of *C. auris* clinical isolates present amino acid substitutions at the *ERG11* gene, which has been associated to fluconazole resistance in other *Candida* spp (Lockhart et al., 2017b).

The search for new antifungal alternatives is of great importance due to the urgent need to solve the problem of resistance in this pathogen. One strategy is drug repurposing which involves finding new applications for drugs that are already available for use. This approach results in a rapid application at a lower cost compared with the development of a new drug (Corsello et al., 2017). In this work, we describe several off-patent molecules that present antifungal activity against *C. auris*.

Using this strategy, we initially found 27 compounds with activity against *C. auris*. Among the active compounds, there were drugs from different therapeutic classes and 13 antifungal drugs (Tables 2, 3). The antifungal activity of seven of them (trifluoperazine dihydrochloride, suloctidil, ciclopirox ethanolamine, ebselen, tamoxifen citrate, thiethylperazine dimalate, pyrvinium pamoate) was confirmed with seven clinical *C. auris* isolates.

One of the most active compounds was ebselen, which is an anti-inflammatory and antioxidant drug currently under clinical trials for the prevention and treatment of various disorders such as cardiovascular diseases, arthritis, stroke, atherosclerosis, and cancer. Ebselen mimics the activity of glutathione peroxidase and in consequence protects against oxidative damage (Azad and Tomar, 2014). However, in prokaryotes, ebselen inhibits the activity of thioredoxin reductase, leading to an increase in the amount of reactive oxygen species in the cell and decreased viability (Azad and Tomar, 2014). In the case of fungi, ebselen is active against FCZ-resistant *C. albicans* strains (Billack et al., 2009) and can behave as fungistatic or fungicidal, depending on the concentrations used in the assays. Furthermore, ebselen is at least 10-fold more potent than fluconazole. These authors suggested that the antifungal activity of ebselen could be due to its interaction with the sulfhydryl group of L-cysteine residues within the plasma membrane H^+^-ATPase (Billack et al., 2009). Other authors have reported that ebselen is active against several fungal species [*C. albicans, C. glabrata, C. tropicalis, C. parapsilosis, Cryptococcus neoformans*, and *C. gattii*] at MICs ranging from 0.015 to 2 mg/L (Thangamani et al., 2017; Eyre et al., 2018). *In vivo* studies using *Caenorhabditis elegans* as model of infection have shown that ebselen activity is superior to FCZ, 5FC, and AmB. Furthermore, in fungi, ebselen depletes intracellular glutathione levels increasing the production of reactive oxygen species (Thangamani et al., 2017). Our findings are in agreement with the recent work by Wall et al., who found that ebselen has antifungal activity against *C. auris* and other *Candida* spp. such as *C. lusitaniae, C. krusei, C. albicans, C. dubliniensis, C. parapsilosis, C. tropicalis*, and *C. glabrata* (Wall et al., 2018).

Suloctidil was another compound with important activity against *C. auris*, with MIC values ranging from 4 to 8 mg/L. Suloctidil is an antiplatelet drug with reported activity against *C. albicans* and *C. neoformans* (Butts et al., 2013). Suloctidil is also effective against *C. albicans* biofilms and inhibits hyphae formation, one of the most important virulence factor of *C. albicans* (Zeng et al., 2017). By using a chemical-genetic profile approach, it was found that suloctidil interferes with membrane trafficking and vacuolar biogenesis (Spitzer et al., 2011).

We also found that the anthelmintic drug pyrvinium pamoate has a noticeable activity against *C. auris*, with MIC values ranging from 1 to 4 mg/L against all the strains tested. Pyrvinium pamoate is synergistic with miconazole against *C. albicans* biofilms (De Cremer et al., 2015). Furthermore, pyrvinium pamoate inhibits the growth of the *C. albicans* FCZ resistant isochromosome 5L strain, which contains two copies of the left arm of the chromosome 5, a mechanism that confers resistance to FCZ (Chen et al., 2015). More recently, it has been shown that pyrvinium pamoate is active against the black yeast *Exophiala dermatitidis* strains and exhibits synergy with ITZ, FCZ, and PSZ (Gao et al., 2018).

Ciclopirox ethanolamine, with MICs values ranging from 0.5 to 1 mg/L also presented activity against *C. auris*. It is thought that it acts by chelating trivalent cations, which induces cell permeability alterations (Gupta and Skinner, 2003). This compound has antifungal activity against several species, including all clinically relevant dermatophytes, molds and yeasts, as well as those with reduced susceptibility to azoles, such as *C. glabrata, C. krusei*, and *C. guilliermondii* (Niewerth et al., 2003).

In addition to the search for new antifungal compounds, another important tool to fight resistant fungal infection is to improve the action of the known antifungal drugs currently used in therapy. One approach is finding molecules or drugs that act synergistically with known antifungal drugs. In example, synergy between micafungin and voriconazole has been found with multiple *C. auris* strains (Fakhim et al., 2017).

We evaluated the synergism activity of three of the screened compounds, ebselen, suloctidil, and ciclopirox ethanolamine with two antifungal drugs, voriconazole and anidulafungin.

Ebselen only showed a moderate synergism with ANID. In our results, ANID at higher concentrations >0.12 mg/L produced a growth inhibition of 60–70%, and the addition of ebselen increased this inhibition to >90%. Wall et al. assayed the synergism of ebselen with FCZ, AmB, and caspofungin against planktonic cells and biofilm from *C. auris* and *C. albicans* and they found that only the combination of ebselen with FCZ was synergic against *C. albicans*. However, none of the combinations showed synergism against *C. auris* (Wall et al., 2018).

The only drug that showed synergism with VCZ was Suloctidil. The combination with suloctidil decreased the MIC values for VCZ from 2–4 to 0.12–1 mg/L. Other authors have found that suloctidil is synergistic with FCZ against *C. neoformans* (Spitzer et al., 2011; Butts et al., 2013). As proposed by Spitzer et al., this combination affects fungal viability in different ways such as increasing fungal cell sensitivity to accumulation of intermediates of the ergosterol pathway, difficulting FCZ export by drug efflux pumps, and inhibiting import of extracellular ergosterol (Spitzer et al., 2011), although these hypothesis have not been fully validated yet.

Our results are in agreement with the recent report by Wall et al. (2018) in which a similar approach was taken to identify several compounds with activity against *C. auris*. Among the drug hits identified by both studies, four compounds were common: suloctidil, ebselen, pyrvinium pamoate and dimethisoquin hydrochloride, suloctidil and ebselen being the most effective compounds against *C. auris* (>80% of inhibition). Wall et al. also showed that ebselen has a broad-spectrum antifungal activity, being also able to inhibit biofilms of *C. auris* and *C. neoformans*. All these data highlight the potential of ebselen as an antifungal drug. Our study also revealed the effectiveness of different drugs from Wall et al. (2018), probably due to the differences in the concentration of the drugs employed in the screening (50 vs. 20 μM) and to the use of different *C. auris* strains.

At this point the *in vivo* use of these drugs cannot be anticipated nor can their associated toxicity, because the concentration required to confer protection against *C. auris* may be different from the one used in current applications. Besides this limitation, our findings indicate that drug repurposing can be a very important tool for finding active compounds against multi-resistant fungal pathogens. In addition, the use of ebselen or suloctidil in combination with ANID or VCZ, respectively, warrants further consideration.

## Data Availability

The datasets generated for this study are available on request to the corresponding author.

## Author Contributions

HdO, MM, SR, EM, and OZ conceived and designed the experiments. HdO, MM, and SR performed the experiments. HdO, SR, EM, and OZ analyzed the data. HdO, MG, JP, AR-G, EM, and OZ drafted the manuscript. All authors read and approved the final manuscript.

### Conflict of Interest Statement

The authors declare that the research was conducted in the absence of any commercial or financial relationships that could be construed as a potential conflict of interest.
